# *Lactobacillus helveticus*: the proteolytic system

**DOI:** 10.3389/fmicb.2013.00030

**Published:** 2013-03-05

**Authors:** M. W. Griffiths, A. M. Tellez

**Affiliations:** Department of Food Science, Canadian Research Institute for Food Safety, University of GuelphGuelph, ON, Canada

**Keywords:** proteolytic, *Lactobacillus*, *helveticus*, milk, proteins

## Abstract

*Lactobacillus helveticus* is one of the species of lactic acid bacteria (LAB) most commonly used in the production of fermented milk beverages and some types of hard cheese. The versatile nature of this bacterium is based on its highly efficient proteolytic system consisting of cell-envelope proteinases (CEPs), transport system and intracellular peptidases. Besides use of *L. helveticus* in cheese processing, the production of fermented milk preparations with health promoting properties has become an important industrial application. Studies have shown that fermented dairy products are able to decrease blood pressure, stimulate the immune system, promote calcium absorption, and exert an anti-virulent effect against pathogens. These beneficial effects are produced by a variety of peptides released during the hydrolysis of milk proteins by the proteolytic system of *L. helveticus*, which provides the bacterium with its nutritional requirements for growth. In recent years, studies have focused on understanding the factors that affect the kinetics of milk protein hydrolysis by specific strains and have concentrated on the effect of pH, temperature, growth phase, and matrix composition on the bacterial enzymatic system. This review focuses on the role of the proteolytic system of *L. helveticus* in the production of bioactive compounds formed during fermentation of dairy products. Taking advantage of the powerful proteolytic system of this bacterium opens up future opportunities to search for novel food-derived compounds with potential health promoting properties.

## Introduction

*Lactobacillus helveticus* is a homofermentative, thermophilic lactic acid bacterium widely used in the manufacture of Swiss type and, Italian aged cheeses, and fermented milk drinks (Gatti et al., 2004; Vinderola et al., 2007a). *L. helveticus* has the ability to reduce bitterness and give characteristic flavor to cheese, which makes this bacterium an important component of starter cultures for the dairy industry. Consumer interest in functional food has motivated researchers to focus on the bioactive compounds formed during fermentation of dairy products by lactic acid bacteria (LAB) such as *L. helveticus* (Matar et al., 2003; Vinderola et al., 2007b; Rattanachaikunsopon and Phumkhachorn, 2010; Wakai and Yamamoto, 2012). Milk proteins are considered to be the most important source of bioactive peptides that can be released through hydrolysis by proteolytic microorganisms such as *L. helvetius* (Meisel and Bockelmann, 1999; Matar et al., 2001; LeBlanc et al., 2002; Fitzgerald and Meisel, 2003; Kenny et al., 2003; de Moreno de LeBlanc et al., 2005). During milk fermentation only 1–2% of milk proteins undergo proteolysis and the principal substrate is casein but limited degradation of whey proteins may also occur (Khalid et al., 1991; Szwajkowska et al., 2011). The effect of this proteolysis is that fermented milks have a higher content of peptides and free amino acids, especially valine, histidine, serine and proline, than non-fermented milk (Matar et al., 2003). LAB rely on their complex proteolytic system to obtain all the necessary free amino acids required for growth in milk. *L. helveticus* strains are among the most nutritionally fastidious LAB as they present multiple amino acid auxothrophies (Hebert et al., 2002). In order to assure its nutritional requirements when grown in milk, *L. helveticus* counts on a potent proteolytic system capable of producing short peptides and liberating amino acids from the casein matrix. This explains why it has higher proteolytic activity than most other lactobacilli and why it hydrolyses more casein in culture media than other species (Savijoki et al., 2006). The proteolytic system of *L. helveticus* and the majority of LAB consists of proteinases which initially cleave caseins to large peptides, peptidases (intracellular), which further degrade these peptides to small peptides and amino acids, and specific transport proteins which transport amino acids and peptides across the cytoplasmic membrane (Kenny et al., 2003).

## Proteolytic system

### Proteinases

The main source of amino acids and peptides produced by LAB are derived from caseins, the most abundant protein in milk (Meisel and Bockelmann, 1999). The hydrolysis of caseins by LAB is initiated by a cell-envelope proteinase (CEP) that degrades the protein into oligopeptides. Moreover, gene deletion studies have shown that LAB are not able to grow in milk in the absence of a functional CEP (Mayo et al., 2010). This enzyme belongs to the family of subtilisin and it is a serine protease. CEPs are anchored to the cell wall via a mechanism involving the typical sortase A (SrtA) (Dandoy et al., 2011). Most of the LAB possess only one CEP but *L. helveticus* has been reported to have at least two: PrtH and PrtH2 (Gilbert et al., 1997). The CEPs of LAB are synthesized as preproteins of ~2000 residues and are composed of seven functional domains: S, PP, PR, A, B, H, and W (Fernandez-Espla et al., 2000). PrtH1 and PrtH2 present in *L. helveticus* differ from the CEPs of other LAB because they lack the anchor domain (AN), which is located at the C-terminus of the CEP sequence. Some authors consider that there are at least two other types of CEP in *L. helveticus*, namely PrtH3 and PrtH4 (Yamamoto et al., 1998; Savijoki et al., 2006; Broadbent et al., 2011; Sadat-Mekmene et al., 2011a). In *L. helveticus* CNRZ32, two genes encoding the CEPs, *prtH*, and *prtH2* have been reported (Sadat-Mekmene et al., 2011b). Two additional putative genes encoding CEPs have been recently detected in *L. helveticus* CNRZ32, *prtH3*, and *prtH4* (Jensen et al., 2009). In another study, an additional gene, *prtH5*, was detected (Smeianov et al., 2007). The presence of several genes encoding the proteolytic system of *L. helveticus* could explain the high efficiency of its proteolytic system. From the four identified genes, only *prtH2* is common to all the characterized strains of *L. helveticus* (Genay et al., 2009). A single gene (*prtH3*) was found in strain DPC4571 (Broadbent et al., 2008), the *prtH2* gene is present in strains CNRZ2 and MI 2359, while LHC2 seems to have just the *prtH4* gene (Jensen et al., 2009). *L. helveticus* CM4, which has a very strong hydrolytic system, has been reported to have three CEP encoded genes: *prtY, prtH2*, and *prtM2* (Wakai and Yamamoto, 2012). A comprehensive study of the proteolytic system of several strains of *L. helveticus* strains using comparative genome hybridization microarray based on *L. helveticus* CNZR 32 was published recently (Broadbent et al., 2011). This study showed that there is a high intraspecific diversity of genes encoding CEPs in the 51 strains of *L. helveticus* analyzed. Twelve percent of the strains tested contained all four CEP paralogs, 8% contained 3 paralogs, whereas 42% had genes for just two CEPs. Among the strains tested the most abundant paralog was *prtH3*, followed by *prtH* and *prtH4* paralogs. *L. helveticus* BGRA43 isolated from human feces has strong proteolytic activity and is able to hydrolyze α_s1_-, β-, and κ-caseins completely (Lozo et al., 2011). Analysis of the proteinase from BGRA43 showed the only active gene was *prtH*. In *L. helveticus* as in other LAB species, CEP activation requires a maturation proteinase termed PrtM. In *L. helveticus* CNRZ32 two PrtMs (PrtM1 and PrtM2) have been identified (Savijoki et al., 2006). Further studies (Genay et al., 2009; Broadbent et al., 2011; Zhao et al., 2011) have reported that PrtM is required for activation of PrtH and PrtM2 plays a role in activation of other CEP paralogs in *L. helveticus*. The presence of the various CEPs in *L. helveticus* strains that have been characterized is presented in Table 1.

**Table 1:** **Cell envelope proteinases and origin of characterize *L. helveticus* strains**.

***L. helveticus* strain**	**Source and/or application**	**CEP**		**References**
		***PrtH***	***PrtH2***	
CNRZ32	Artisanal starter used in Comté, cheese starter used to reduce bitterness	+	+	Pederson et al., 1999; Dudley et al., 1996
CNRZ32JS	Artisanal starter used in Comté	+	+	Genay et al., 2009
CNRZ67	Artisanal lactic starter of Gruyère de Comté	−	+	Genay et al., 2009
CNRZ240	Artisanal starter of Gruyère de Comté	−	+	Genay et al., 2009
CNRZ241	Artisanal starter used in Comté	+	+	Genay et al., 2009
CNRZ303	Artisanal starter used in Comté and Swiss cheese	−	+	Deutsch et al., 2003; Zevaco and Gripon, 1988
CNRZ328	Artisanal starter of Emmental; Prt^−^ phenotype	−	+	Hebert et al., 2002
CNRZ414	Cow milk Koumiss	−	+	Genay et al., 2009
CNRZ450	Commercial starter of Emmental	−	+	Genay et al., 2009
CNRZ498	Yogurt	−	+	Genay et al., 2009
CNRZ762	Artisanal starter of Gruyère de Comté	−	+	Genay et al., 2009
CNRZ891	Commercial starter	−	+	Genay et al., 2009
CNRZ1080	Not determined	−	+	Genay et al., 2009
CNRZ1095	Not determined	−	+	Genay et al., 2009
CNRZ1111	Commercial starter	−	+	Genay et al., 2009
CNRZ1317	Commercial starter used in Tallegio	−	+	Genay et al., 2009
ISLC5	Artisanal lactic starter of Grana Padano	−	+	Valence and Lortal, 1995
CP615	Not determined	−	+	Genay et al., 2009
CP790	Starter for cultured milk products	−	+	Yamamoto et al., 1993
CP53		−	+	Ono et al., 1997
CIP 103146T	Artisanal starter used in Emmental	−	+	Genay et al., 2009
CRL1062	Argentinean hard cheese	−	+	Hebert et al., 1999; Christiansen et al., 2007
CRL581	Argentinean cheese	−	+	Hebert et al., 1997
ROSELL 5088	Not determined	+	+	Genay et al., 2009
ROSELL 5089	Not determined	+	+	Genay et al., 2009
DPC4571	Swiss cheese whey	−	+	Slattery et al., 2010
ITG LH1	Whey	+	+	Deutsch et al., 2000
ITG LH2	Aminopeptidase-deficient mutant strain derived from ITG LH1	−	+	Blanc et al., 2003
ITG LH3	Aminopeptidase-deficient mutant strain derived from ITG LH1	+	+	Blanc et al., 2003
ITG LH77	Artisanal starter, commercial strain	−	+	Deutsch et al., 2000
L89	Starter for production of Grana cheese	−	+	Martin-Minervini et al., 1994
PR4	Isolated from Italian cheese	−	+	Minervini et al., 2003; Di Cagno et al., 2006
ZUC2	Grana Padano cheese	−	+	Scolari et al., 2006

### Peptide and amino acid transport system

The second stage of protein degradation is the transport of di-, tri-, and oligo-peptides into the cell by different peptide transport systems. One of the first publications on the peptide transport system of *L. helveticus* concluded that Leu, Ile, Val, Thr, and Lys were transported by a secondary transport system, whereas the amino acids Asp, Glu, His, Arg, and Tyr seemed to be transported by an ATP-driven system (Nakajima et al., 1998). After *L. helveticus* strains DPC4571 (Callanan et al., 2008) and H10 (Zhao et al., 2011) were sequenced and information regarding their transport system became available, it was determined that strain DPC4571 possesses three peptide transport systems, the oligopeptide Opp transport system and the di- and tri-peptide transport system, Dpp and DtpT, respectively. Conversely, H10 has two peptide transport systems, the Opp and dtpT systems. These results indicate that the proteolytic system may differ between different strains in *L. helveticus*.

### Peptidases

Peptidases are a very important part of the proteolytic system in LAB since they are involved in the hydrolysis of peptides to release essential amino acids (Christensen et al., 1999). Up until 2003 just 11 peptidases were known to be part of the proteolytic system of *L. helveticus*: two proline-specific endopepeptidases, PepE and PepO; a tripeptidase PepT; four aminopeptidases, PepX, PepI, PepQ, and PepR; and four dipeptidases, PepD, PepV, PepC, and PepN (Christensen et al., 1999; Savijoki and Palva, 2000; Kenny et al., 2003; Pan and Tanokur, 2004). Recently it was reported that strain CNRZ32 has seven oligoendopeptidases, three general aminopeptidases, five proline-specific peptidases, eight di- or tri-peptidases, and at least six other peptidases (Broadbent et al., 2011).

The proteolytic system of *L. helveticus* CNRZ32 has been widely studied (Khalid and Marth, 1990; Fernández et al., 1994; Shao et al., 1997; Chen et al., 2003; Christensen and Steele, 2003; Kilpi et al., 2007) due to its ability to reduce cheese bitterness (Fernández et al., 1994) and to release peptides during milk fermentation with angiotensin converting enzyme (ACE) inhibitory activity (Kilpi et al., 2007). It is well known that peptides derived from β-casein (f193–202) and α_−s1_ (f1–9) have been implicated in the development of bitterness in cheese (Christensen et al., 2003). For this reason, research has focused on studying the *L. helveticus* endopeptidases that are responsible for the hydrolysis of these caseins (Chen et al., 2003; Christensen et al., 2003). The endopeptidase PepO2 plays a pivotal role in the ability of *L. helveticus* CNRZ32 to reduce bitterness because of its specificity for bonds containing proline. The peptidase PepDA was not necessary for the liberation of essential amino acids since the deletion of the gene *pepDA* had no effect on the growth of strain CNRZ32 (Dudley et al., 1996). Similarly, the dipeptidase with proline activity (PepR) has been found not to be a limiting enzyme for growth of CNRZ32 (Shao et al., 1997). A recent study on the amino peptidase activity of six strains of *L. helveticus* grown in milk or MRS (de Man, Rogosa, and Sharpe) medium reported that the mechanism of regulation depended on the specific peptidase and possibly on the specific strain. Higher aminopeptidase activity against almost all substrates used for the study was found after growth in milk (Jensen and Ardo, 2010). The effect of amino-peptidases PepN and PepX present in *L. helveticus* CNRZ32 on the production of peptides with antihypertensive activity has been also a subject of study. Milk fermented with peptidases-negative mutants, induced higher production of ACE-inhibitory activity compared with milk fermented with wild-type strains (Kilpi et al., 2007). The importance of peptidases PepC, PepN, and PepX for the growth of *L. helveticus* CNRZ32 was evident when a multi-peptidase deletion mutant (*pepC*^−^, *pepN*^−^, and *pepX*^−^) was studied. The growth rate inhibition in milk exhibited by the mutants was overcome by supplementation with amino acids associated with the peptidase that was absent in each of the mutants (Christensen et al., 2003).

The essential role that *L. helveticus* peptidases have in the manufacture of Swiss cheese was confirmed when the proteolytic systems of selected LAB were compared (Deutsch et al., 2000). Peptidases of the *L. helveticus* strains tested showed higher proteolytic activity as reported previously (Valence et al., 1998, 2000) and the presence of one carboxypeptidase in all the strains tested was confirmed. An endopeptidase with the ability to hydrolyze peptides Val-Pro-Pro-Phe-Leu and Ile-Pro-Pro-Leu-Thr to produce Val-Pro-Pro and Ile-Pro-Pro, respectively, was isolated and characterized from *L. helveticus* CM4 (Ueno et al., 2004). Based on the results, it was suggested that the enzyme is a key player in the hydrolysis of the C-terminal during the production of Val-Pro-Pro and Ile-Pro-Pro in fermented milk. The highest aminopeptidase activity for all the strains was observed for the substrates Arg-, and Lys-pNA.

## Bioactive peptides produced from hydrolysis of milk proteins

Today, milk proteins are considered the most important source of bioactive peptides and an increasing number of bioactive peptides have been identified in milk protein hydrolysates and fermented dairy products (Clare et al., 2003; Silva and Malcata, 2005; Korhonen and Pihlanto, 2006). Milk can be the source of peptides with different bioactivities with potential interaction and synergism among them as well as with other non-peptide components. Upon oral administration, bioactive peptides may affect the major body systems. For this reason, the potential of distinct dietary peptide sequences to promote human health by reducing the risk of chronic diseases or boosting natural immune protection has aroused a great deal of scientific interest over the past few years (Schanbacher et al., 1998). Bovine milk contains 32 g/L of proteins, of which 80% are caseins and 20% are whey proteins. Casein, in addition to being a very efficient calcium and phosphate transport for the newborn, hides a series of bioactive molecules in its sequences. The caseins are a family of phosphoproteins and they have been designated α_s1_-, α_s2_-, β-, and κ-caseins, with α_s1_ being the major protein found in bovine milk. So far, no specific physiological property has been proposed for the whole casein system, whereas various peptides that are inactive in the amino-acid sequence have been proven to possess bioactivities (Pihlanto-Leppala et al., 1999; Florisa et al., 2003; Kilara and Panyam, 2003). CEPs of *L. helveticus* are highly specific since the sites of cleavage on the caseins differ from one strain to another depending on the type of casein (α- or β-casein) (Zevaco and Gripon, 1988; Jensen et al., 2009; Sadat-Mekmene et al., 2011a). A study conducted on the proteolytic system of *L. helveticus* Zuc2, reported the potential presence of two CEPs (Scolari et al., 2006). The most loosely bound CEP was partially characterized, which was able to hydrolyze primarily β-casein and to a lesser extent κ- and α_s1_-casein. The CEP cleaved α_s1_-CN (f1–23) in contrast with other CEPs from various *L. helveticus* strains. The proteolytic system of fifteen strains of *L. helveticus* more efficiently hydrolyzed β-casein than α_s1_-casein (Sadat-Mekmene et al., 2011b). Moreover, among all the tested strains there were no differences in the extent and time of hydrolysis of β-casein in strains with one CEP or more. Different studies have agreed that CEPs of *L. helveticus* can cleave α_s1_- or β-casein at different sites not specifically related to one type of amino acid (Zevaco and Gripon, 1988; Broadbent et al., 2011; Sadat-Mekmene et al., 2011b). For instance, strains *L. helveticus* CNRZ 303 and CP790 hydrolyzed several bonds at the C-terminal of β-casein, while strains CNRZ 32, CNRZ 303 and LHC2 hydrolyzed β-casein at the N-terminal (Jensen et al., 2009). Some strains of *L. helveticus* such as CP790 (Yamamoto et al., 1994), CP53 (Ono et al., 1997), and PR4 (Di Cagno et al., 2006) are able to hydrolyze both α_s1_- and β-casein. Two comprehensive studies have concluded that the hydrolysis of α_s1_-casein by *L. helveticus* strains predominantly occurs at the N-terminal (Jensen et al., 2009; Sadat-Mekmene et al., 2011a). However, it is clear that most of the characterized strains of *L. helveticus* are able to extensively hydrolyze β-casein. The CEPs of *L. helveticus* are also able to hydrolyze κ-casein and α_s2_-casein; although to a lesser extent than for the other caseins (Ezzat et al., 1985; Yamamoto et al., 1998; Scolari et al., 2006; Jensen et al., 2009; Sadat-Mekmene et al., 2011a).

Whey proteins have a globular structure and are more water-soluble than caseins. The principle fractions are β-lactoglobulin, α-lactalbumin, bovine serum albumin (BSA), and immunoglobulins (Ig). In the past, whey was considered a waste product or a raw material for relatively low-value commodities. However, in the last decade with the emergence of new separation technologies, the potential of whey proteins as bioactive compounds has been realized (Chatterton et al., 2006).

Production of bioactive peptides from enzymatic hydrolysis of whey proteins has been reviewed (Pihlanto-Leppala, 2001; Madureira et al., 2010). The opioid peptides, α-lactorphin and β-lactorphin were released during *in vitro* proteolysis of whey proteins and whey hydrolysates showed ACE-inhibitory activity after proteolysis with digestive enzymes, and several active peptides (Pihlanto-Leppala, 2001). To date, there are few publications that have focused on the production of bioactive peptides released during fermentation of whey with LAB. *L. helveticus* LH-2 was able to hydrolyze α-lactalbumin and release novel bioactive peptides with immunomodulatory properties (Tellez et al., 2010). Whey protein-based media have been used as a fermentation substrate for *L. acidophilus* La-5 and bioactive molecules produced during its growth are able to down-regulate genes involved in encoding the Type 3 Secretion System of *E. coli* O157:H7 (Medellin-Pena et al., 2007; Medellin-Pena and Griffiths, 2009; Zeinhom et al., 2012) and *Salmonella* (Bayoumi and Griffiths, 2012).

## Physiological role of the bioactive compounds generated by *L. helveticus*

Traditionally milk proteins have been used as a raw material to obtain bioactive peptides because it is considered to be safe and relatively inexpensive. The production of bioactives in fermented milk and cheese has been widely studied (Leclerc et al., 2002; Matar et al., 2003; Gómez-Ruiz et al., 2004a,b; Fitzgerald and Murray, 2006; Jensen et al., 2009; López-Expósito et al., 2012). Upon oral administration, these bioactive peptides may have an impact on various functions in the human body depending on their amino acid sequence (Korhonen and Pihlanto, 2006). Various peptides with physiological functions, such as immunostimulating peptides, antimicrobial peptides, opioid peptides, mineral binding peptides and antihypertensive peptides have been isolated from products fermented with *L. helveticus*.

### Antihypertensive

Antihypertensive peptides have been extensively reviewed (Ono et al., 1997; Yamamoto et al., 1998; Wakai and Yamamoto, 2012).

The ability of *L. helveticus* to produce antihypertensive peptides has been attributed to the CEP (Ono et al., 1997; Wakai and Yamamoto, 2012). To date, no relationship has been established between the antihypertensive effect and proteinase specificity. A number of researchers have concluded that the functional properties of *L. helveticus* depend on the specific strain (Slattery et al., 2010). Thus, strains CM4, CNRZ32, and DPC4571 that are able to hydrolyze milk proteins to produce antihypertensive peptides differ slightly in terms of their CEP genes when compared with strains that are incapable of producing them (Sadat-Mekmene et al., 2011b; Wakai and Yamamoto, 2012).

### Immunomodulatory activity

Milk fermented with *L. helveticus* can enhance specific and non-specific immunity (Laffineur et al., 1996; LeBlanc et al., 2004; Vinderola et al., 2007a,b; Tellez et al., 2010, 2011). Milk fermented with *L. helveticus* R389 was able to induce immune response and exert a protective effect against *Salmonella* Typhimurium and *E. coli* O157 (LeBlanc et al., 2004; Vinderola et al., 2007b). The positive effect on the mucosal and tumoral immunity was directly related to the proteolytic system of *L. helveticus* R389, since milk containing the non-proteolytic variant was not able to instigate an immune response (Matar et al., 2001). Although *L. helveticus* R389 is able to hydrolyze milk proteins to produce bioactive peptides that have a beneficial effect on the immune system, not much work has been done to characterize the components of the proteolytic system that is responsible for generation of these peptides, nor on the identification of the peptides responsible for the immunomodulatory effect. *In vitro* experiments have shown that milk fermented with *L. helveticus* LH-2 can enhance cellular immune responses (Ng and Griffiths, 2002; Tellez et al., 2010) and have a protective effect against *Salmonella* infection *in vivo* (Tellez et al., 2011). Four novel bioactive peptides derived from β-casein and α-lactalbumin following fermentation of milk with *L. helveticus* LH-2 were identified (Tellez et al., 2010). Three of the peptides share a common amino acid sequence with β-casein with variation at N- and C-terminals (f 148–154, f 145–160, and f 143–154). Peptides identified have in their structure a high percentage of proline and also contain residues of lysine and histidine.

The S-layer protein extract of *L. helveticus* R0052 has been shown to ameliorate the pathogenesis of *E coli* O157:H7 (Sherman et al., 2005; Johnson-Henry et al., 2007). Epithelial cells treated with S-layer protein extract prior to infection with *E. coli* O157 maintained their cellular integrity and barrier function. Beganovic et al. (2011) isolated and characterized the S-layer from *L. helveticus* M92 and tested its functionality *in vivo*. The *slpA* gene encoding the S-layer protein was sequenced and compared with other probiotic bacteria. The purified S-layer protein enhanced the immunomodulatory response in *Salmonella*-infected mice to a greater extent than *L. helveticus* M92 cells after the S-layer protein was removed.

The antimutagenic effect of milk fermented with *L. helveticus* L89 was attributed to the proteolytic system of this strain, since the non-proteolytic variant (Prt^−^) did not have the same effect (Matar et al., 1997).

### Anti-cancer

An *in vivo* study using a model of breast cancer in mice concluded that milk fermented with *L. helveticus* R389 or *L. helveticus* L89 delayed tumor development (de Moreno de LeBlanc et al., 2005). Modulation of cytokine production and apoptosis of tumor cells were observed in mice fed with fermented milk, which were related to the potent proteolytic ability of strains used in this study especially R389. In the same study, milk fermented with a proteolytic-deficient variant of *L helveticus* L89 did not exert the same regulatory response observed in mice fed with milk fermented with R389 or L89.

### Calcium binding

Recently, several strains of *L. helveticus* were tested to evaluate their calcium binding ability. Supernatant from milk fermented with *L. helveticus* LA, a highly proteolytic strain, exerted a high Ca-binding activity (Dimitrov, 2009). A peptide produced from the hydrolysis of alpha casein with A1 responsible for this effect was purified and sequenced.

*L. helveticus* 16H and LBK-16H have been reported to have a positive effect on calcium metabolism, bone volume, bone formation and bone mineral density (Narva et al., 2004a,b,c, 2007). An *in vitro* study (Narva et al., 2004b) on the effect of milk fermented with *L. helveticus* LBK-16H and its isolated peptides [isoleucyl-prolylproline (IPP) and valyl-prolyl-proline (VPP)] exerted an anabolic effect on bone. Milk fermented with *L. helveticus* 16H was also shown to increase bone mineral density and bone mineral content in growing rats (Narva et al., 2004a). The same strain has been reported to produce some bioactive peptides in milk, which exert a positive effect on calcium metabolism (Narva et al., 2004c). To test the effect of milk fermented with *L. helveticus* LBK-16H and synthetic peptide VPP, a study on ovariectomized rats was conducted (Narva et al., 2007). Fermented milk was able to prevent bone loss while VPP dissolved in water did not prevent ovariectomy-induced bone loss. Perhaps the synergies between different components produced during fermentation could be responsible for the functionality of fermented milk.

Table 2 presents a summary of the *L. helveticus* strains that have been reported to produce bioactive peptides from milk proteins.

**Table 2:** **Bioactive peptides produced from hydrolysis of milk proteins by *L. helveticus* strains**.

**Bioactivity**	**Strain**	**References**
Antihypertensive	CP790	Wakai and Yamamoto, 2012
	CP611	
	CP615	
	JCM1006	
	JCM1004	
	CNRZ32	Kilpi et al., 2007
	CM4	Ueno et al., 2004
	R211	Leclerc et al., 2002
	R389	Leclerc et al., 2002
	LMG 11474	Vermeirssen et al., 2003
	LBK-16H	Seppo et al., 2003
Immunomodulatory	5089	Laffineur et al., 1996
	389	Matar et al., 2001; LeBlanc et al., 2004; Vinderola et al., 2007a,b
	LH-2	Ng and Griffiths, 2002; Tellez et al., 2010, 2011
	R0052	Sherman et al., 2005; Johnson-Henry et al., 2007
	M92	Beganovic et al., 2011
	L89	Matar et al., 1997
	L89	de Moreno de LeBlanc et al., 2005
Calcium binding	16H	Narva et al., 2004a,b,c, 2007
	LBK-16H	
	A1	Dimitrov, 2009

## Effect of the growth medium on the *L. helveticus* proteolytic system

Even though there are a number of studies that have investigated the proteolytic ability of LAB, little is known about the effect of the growth media on their proteolytic system. It is well known that when milk is used as a growth media for LAB, their proteolytic system is activated (Wakai and Yamamoto, 2012). This is caused by the limited amount of amino acids present in milk and the nutritional needs of LAB. One of the few studies done on the modulation of CEPs by peptide supply (Hebert et al., 2002), concluded that the proteinase activity of the LAB strains used in this study was reduced in cells grown in a peptide-rich medium, such as MRS. Inparticular, the activity of *L. helveticus* CRL 1177 showed a 100-fold reduction in enzyme activity when cells were grown in MRS. Conversely, the peptidase activity of the *L. helveticus* strains tested were not affected when grown in a peptide-rich medium. These results agreed with previous findings of this research group (Hebert et al., 2000). Another study conducted on the effect of *L. helveticus* R0052 to inhibit *E coli* O157:H7 attachment (Johnson-Henry et al., 2007), tested the influence of different media on the S-layer protein. The authors concluded that, even though there was no differences in the structural formation of the S-layer, the physiological differences between bacteria grow under different conditions are unknown. Smeianov et al. (2007) showed that *L. helveticus* CNRZ 32 genes encoding CEPs, endopeptidases and oligopeptide transporters were upregulated when the strain was grown in milk compared with the levels of expression in MRS medium. In another study (Jensen et al., 2009), CEP activity of six *L. helveticus* strains on α_s1_- and β-casein in MRS and milk was evaluated. The CEPs of the different strains hydrolyzed intact α_s1_- and β-casein after growth in milk, but not in MRS.

The cell extract of six *L. helveticus* strains were analyzed for aminopeptidase activity (Jensen and Ardo, 2010). It was concluded that the peptidase activities were generally higher in the *L. helveticus* strains after growth in skim milk than in MRS, with the exception of Pro-pNA, which was affected by the growth medium in a strain dependent manner. Other studies on the peptidase activity of DPC 4571 and M10 in different growth media showed similar results (Kenny et al., 2003; Simova and Beshkova, 2007). The regulation of genes encoding peptidases of *L. helveticus* CNRZ 32 have been studied by Smeianov et al. (2007), who observed an up-regulation of the *pepI* gene during growth in MRS, up-regulation of the genes pepN, pepX, and pepR during growth in skim milk, and the same level of expression of pepC was seen in skim milk and MRS. The impact of the growth medium on proteinase activity was evident in a study conducted on *L. helveticus* CRL581 (Hebert et al., 1997). Proteinase activity decreased in the presence of an available nitrogen source and showed an increment of activity when the growth medium was milk.

## Conclusions

Traditionally *L. helveticus* has been considered to be a very important bacterium for cheese manufacture because its ability to improve cheese quality. In the last 20 years it has became even more relevant due to its ability to produce bioactive compounds during milk fermentation. The industrial importance of *L. helveticus* depends on its effective proteolytic system, which seems to be the most efficient between LAB, as well as the specificity of different strains. We have gained some knowledge of the proteolytic system of this bacterium but there are still aspects that require further study. For instance, little is known regarding the specificity of the CEP on caseins, the transport system of the peptides, and the factors that can affect both the individual components of the system and the proteolytic system as a whole. Variations in the proteolytic system of different strains might not be a pivotal factor for growth of the *L. helveticus* but might have a big impact on the production of bioactive peptides. With the genetic and biochemical tools available it is possible to manipulate the proteolytic system, which can lead to production of bioactive peptides of particular interest or alternatively enzymes isolated from suitable microorganisms can be used alone. Technological challenges in cheese production can be overcome by gaining a deeper understanding of the kinetics of milk hydrolysis by *L. helveticus*. More research is necessary to shed light on the different components of the proteolytic system of *L. helveticus* and other LAB and their role in the production of health promoting compounds from milk fermentation.

### Conflict of interest statement

The authors declare that the research was conducted in the absence of any commercial or financial relationships that could be construed as a potential conflict of interest.
